# Implications of pacemaker implantation after aortic valve surgery for endocarditis: a nationwide study^†^

**DOI:** 10.1093/ejcts/ezaf125

**Published:** 2025-04-08

**Authors:** Lisa O F Bearpark, Michael Dismorr, Anders Franco-Cereceda, Ulrik Sartipy, Natalie Glaser

**Affiliations:** Department of Molecular Medicine and Surgery, Karolinska Institutet, Stockholm, Sweden; Department of Molecular Medicine and Surgery, Karolinska Institutet, Stockholm, Sweden; Department of Cardiothoracic Surgery, Karolinska University Hospital, Stockholm, Sweden; Department of Molecular Medicine and Surgery, Karolinska Institutet, Stockholm, Sweden; Department of Cardiothoracic Surgery, Karolinska University Hospital, Stockholm, Sweden; Department of Molecular Medicine and Surgery, Karolinska Institutet, Stockholm, Sweden; Department of Cardiothoracic Surgery, Karolinska University Hospital, Stockholm, Sweden; Department of Molecular Medicine and Surgery, Karolinska Institutet, Stockholm, Sweden; Department of Cardiology, Stockholm South General Hospital, Stockholm, Sweden

**Keywords:** Endocarditis, Valve surgery, Aortic valve, Pacemaker, Outcomes, Long-term survival

## Abstract

**OBJECTIVES:**

Infective endocarditis (IE) is associated with a high risk of atrioventricular block and surgery adds to the risk of receiving a permanent pacemaker. The clinical impact of pacemaker implantation in IE patients is insufficiently studied. Our objective was to analyse long-term clinical outcomes in patients who receive a permanent pacemaker after IE surgery.

**METHODS:**

We conducted a nationwide observational cohort study, including all patients undergoing surgery for aortic valve IE in Sweden 1997–2022. The exposure group was patients who received a permanent pacemaker within 30 days of surgery. We used inverse probability of treatment weighting to account for inter-group differences and flexible parametric models to estimate hazards and cumulative incidences. Outcomes were all-cause mortality, heart failure and reinfection in the prosthetic valve.

**RESULTS:**

Among 2175 patients who underwent surgery for aortic valve endocarditis, 168 (8%) received a permanent pacemaker. The mean age was 59 years; 18% were female. During a mean follow-up of 8.0 years (maximum 26 years), 822 patients (38%) died. At 15 years, the cumulative incidence of all-cause mortality was 60% versus 50% in the pacemaker versus the no pacemaker group; for heart failure, it was 21% versus 16%. We found no association of pacemaker implantation with mortality [hazard ratio (HR) 1.17; 95% confidence interval (CI) 0.86–1.58], heart failure (HR 1.42; 95% CI 0.89–2.29) or reinfection (HR 0.85; 95% CI 0.50–1.45).

**CONCLUSIONS:**

Pacemaker implantation after surgery for aortic valve IE is common but was not associated with an increased risk of death, heart failure or reinfection. Although pacemaker need suggests more advanced disease, these results show that lifesaving surgery is not importantly jeopardised by the need for a pacemaker.

## GRAPHICAL ABSTRACT

**Figure:**
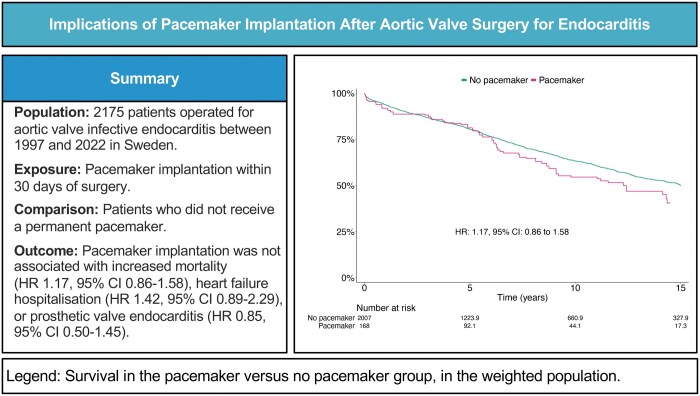

## INTRODUCTION

Infective endocarditis (IE) is a rare, but often deadly disease, which causes structural damage to the heart valves. In many cases, the disease not only affects the valve cusps but also invades the annulus and surrounding tissue. Therefore, disturbances to the conduction system are common in IE patients and the risk of atrioventricular block is high, especially when the aortic valve is involved [1]. Surgery for aortic valve IE requires radical debridement of all infected and necrotic tissue as well as placement of sutures to anchor the replacement valve, which further increases the risk of conduction system disturbances. Out of all IE cases, surgery is necessary in approximately 25–50% [2–4]. Indications for surgery include severe heart failure, high risk of embolism and uncontrolled infection despite adequate antibiotics [5].

Patients undergoing valve surgery for IE run a 7–15% risk of needing permanent pacemaker implantation in the postoperative period, with even higher rates for more extensive IE infections and prosthetic valve IE patients [6–8]. In contrast, surgical aortic valve replacement for non-IE indications leads to pacemaker implantation in 2–6% of patients [9–12]. Associations between pacemaker implantation and adverse outcomes, including death, heart failure, endocarditis and lead-related complications, have been found in studies of surgical aortic valve replacement for primarily non-IE indications [9, 12]. Still, despite a higher rate of pacemaker implantation in patients with IE, the long-term implication of pacemaker placement in these patients remains insufficiently studied. We hypothesized that permanent pacemaker implantation after surgery for endocarditis would also be associated with worse clinical outcomes. Therefore, we investigated the association between long-term clinical outcomes and permanent pacemaker implantation following surgery for aortic valve IE.

## PATIENTS AND METHODS

### Study design

This was a nationwide, population-based, observational cohort study conducted with the approval of the Swedish Ethical Review Authority (dnr: 2019–04131; amendment dnr: 2020–04967). The requirement for informed consent was waived. The Strengthening the Reporting of Observational Studies in Epidemiology (STROBE) guidelines were followed for the reporting of the study [13].

### Setting

This study encompassed all of Sweden’s 8 centres for cardiac surgery. Follow-up for death ended 16 March 2023. Follow-up for heart failure hospitalization and reinfection ended 31 December 2022.

### Data sources

Data were sourced through a cross-linking of Swedish health registers. The Swedish Web-System for Enhancement and Development of Evidence-based Care in Heart Disease Evaluated According to Recommended Therapies (SWEDEHEART) register [14, 15] was used to identify the cohort and baseline characteristics. The Swedish National Patient Register [16] and the longitudinal integrated database for health insurance and labour market studies register [17] were used to obtain additional baseline characteristics. Supplementary Material, Table S2, contains definitions of comorbid conditions. The Swedish Total Population Register was used for information on vital status and date of death [18]. All Swedish citizens and persons, treated in the Swedish healthcare system, have a unique identifying number, allowing for cross-linking of data [19]. We identified IE status using the International Classification of Diseases, Ninth Revision code 421 and Tenth Revision codes I33.0, I33.9, I38.9, I39.8 and B37.6 from the National Patient Register. Exposure was defined as first-time implantation of a permanent pacemaker or implantable cardioverter defibrillator (ICD) within 30 days of surgery, as identified by Nordic Medico-Statistical Committee Classification of Surgical Procedures [20] FPE00, FPE10, FPE20, FPE26, FPF00, FPF10, FPF20, FPG10, FPG20, FPG30, FPG33 and FPG36. Supplementary Material, Table S3, contains a list of the different types of pacemakers implanted, including the number of patients for each type.

### Study population and exposure

We included all consecutive patients who underwent aortic valve replacement for IE in Sweden between 1997 and 31 July 2022. Patients who died within 30 days or had a pacemaker/ICD implanted prior to surgery were excluded. Patients with prosthetic valve endocarditis and concomitant surgeries were included. Patients were separated into 2 groups according to exposure: the pacemaker group and the no pacemaker group.

### Outcomes

All-cause mortality was obtained from the Total Population Register [18]. Heart failure hospitalization and reinfection were obtained from the National Patient Register; Supplementary Material, Table S1, contains a list of the ICD codes used [16]. For all-cause mortality, each patient contributed with follow-up time (in days) beginning from the date of surgery plus 30 days (end of the exposure time), until the date of death. For heart failure, follow-up began on the date of surgery plus 90 days, and for reinfection, follow-up began from the date of surgery plus 120 days. The start of follow-up for heart failure and reinfection diagnosis was based on what made sense given the technical features of each register.

### Statistical analysis

Baseline characteristics are presented as frequencies and percentages for categorical values and means and standard deviations for continuous variables. Inverse probability of treatment weighting using optimization-based weights was used to obtain balanced groups, accounting for differences in baseline characteristics between the pacemaker and no pacemaker groups [21, 22]. All variables in Table 1 as well as the hospital were included in the weighting model and the covariate balance between the groups before and after weighting is shown in Supplementary Material, Fig. S1. Missing data were handled by constructing weights to balance the groups in terms of the missing data. The absolute mean difference was used to evaluate the balance between the groups after matching. An absolute mean difference of less than 10% was considered an ideal balance [23]. Median follow-up time was estimated using the reverse Kaplan–Meier estimator [24]. Survival was estimated using weighted Kaplan–Meier. To estimate the cumulative incidence of heart failure and reinfection, we used weighted flexible parametric survival models. We also constructed a weighted flexible parametric survival model to estimate the mortality hazard. To account for the competing risk of death, a Markov multi-state transition matrix using either the heart failure or the reinfection model together with the mortality model was used, as implemented in the rstpm2 R-package. A 2-sided *P*-value <0.05 was considered statistically significant. Data management and statistical analyses were performed using R statistical software version 4.3.1(R Foundation for Statistical Computing, Vienna, Austria), and Stata statistical software version 18.0 (Stata Corp. LP., College Station, TX, USA); it included the use of the stpm2 and standsurv commands, as well as the WeightIt [25], cobalt [26] survival and rstpm2 R packages.

**Table 1:** Baseline characteristics of 2175 patients who underwent aortic valve replacement for infective endocarditis in Sweden between 1997 and 2022

Variable	Overall	No pacemaker	Pacemaker	*P*-value
No.	2175	2007 (92.3)	168 (7.7)	
Age (years), mean (SD)	59.0 (14.7)	58.6 (14.7)	64.0 (12.7)	<0.001
Female sex	397 (18.3)	370 (18.4)	27 (16.1)	0.511
Non-Nordic birth region	186 (8.6)	175 (8.7)	11 (6.5)	0.410
Education (years)				0.842
<10	657 (30.7)	608 (30.8)	49 (29.7)	
10–12	951 (44.5)	879 (44.6)	72 (43.6)	
>12	530 (24.8)	486 (24.6)	44 (26.7)	
Household income				0.432
Q1 (lowest)	543 (25.0)	509 (25.4)	34 (20.2)	
Q2	543 (25.0)	497 (24.8)	46 (27.4)	
Q3	542 (25.0)	501 (25.0)	41 (24.4)	
Q4 (highest)	542 (25.0)	495 (24.7)	47 (28.0)	
Married	1012 (46.6)	920 (46.0)	92 (54.8)	0.034
Body mass index (kg/m^2^)				0.963
<18.5	47 (2.5)	43 (2.5)	4 (2.6)	
18.5–25	823 (43.8)	755 (43.7)	68 (44.7)	
>25	1008 (53.7)	928 (53.8)	80 (52.6)	
eGFR (ml/min/1.73 m^2^)				0.078
<15	99 (4.9)	96 (5.1)	3 (1.9)	
15–29	85 (4.2)	77 (4.1)	8 (5.2)	
45–59	242 (11.9)	220 (11.7)	22 (14.2)	
30–44	173 (8.5)	153 (8.2)	20 (12.9)	
>60	1431 (70.5)	1329 (70.9)	102 (65.8)	
Diabetes mellitus	323 (14.9)	296 (14.7)	27 (16.1)	0.726
Peripheral vascular disease	43 (2.0)	39 (1.9)	4 (2.4)	0.918
Prior stroke	357 (16.4)	329 (16.4)	28 (16.7)	1.000
COPD	115 (5.3)	106 (5.3)	9 (5.4)	1.000
Alcohol dependence	186 (8.6)	171 (8.5)	15 (8.9)	0.970
Hepatic disease	77 (3.5)	74 (3.7)	3 (1.8)	0.287
Preoperative bleeding event	405 (18.6)	380 (18.9)	25 (14.9)	0.233
History of cancer	302 (13.9)	280 (14.0)	22 (13.1)	0.848
Hyperlipidemia	327 (15.0)	296 (14.7)	31 (18.5)	0.239
Hypertension	857 (39.4)	776 (38.7)	81 (48.2)	0.019
Prior heart failure	596 (27.4)	545 (27.2)	51 (30.4)	0.421
Prior myocardial infarction	171 (7.9)	157 (7.8)	14 (8.3)	0.931
Prior PCI	96 (4.4)	90 (4.5)	6 (3.6)	0.720
Prior atrial fibrillation	480 (22.1)	430 (21.4)	50 (29.8)	0.016
Prior endocarditis	155 (7.1)	144 (7.2)	11 (6.5)	0.883
LVEF				0.078
<30%	65 (3.5)	55 (3.2)	10 (6.6)	
30–50%	520 (27.6)	482 (27.9)	38 (25.0)	
>50%	1296 (68.9)	1192 (68.9)	104 (68.4)	
Prior heart surgery	452 (24.0)	401 (23.1)	51 (33.6)	0.005
Emergent operation	269 (14.3)	251 (14.5)	18 (11.8)	0.439
CABG	215 (9.9)	199 (9.9)	16 (9.5)	0.977
Mitral	396 (18.2)	355 (17.7)	41 (24.4)	0.039
Prosthetic valve endocarditis	435 (20.0)	383 (19.1)	52 (31.0)	<0.001
Ascending aortic surgery	301 (13.8)	268 (13.4)	33 (19.6)	0.031
Tricuspidalis	72 (3.3)	59 (2.9)	13 (7.7)	0.002
Bioprosthetic valve	1313 (60.4)	1191 (59.3)	122 (72.6)	0.001
Root replacement	303 (13.9)	269 (13.4)	34 (20.2)	0.019
Period of surgery (years)				0.006
1997–2005	487 (22.4)	463 (23.1)	24 (14.3)	
2006–2014	797 (36.6)	739 (36.8)	58 (34.5)	
2015–2022	891 (41.0)	805 (40.1)	86 (51.2)	
Hospital				0.013
1	366 (16.8)	348 (17.3)	18 (10.7)	
2	443 (20.4)	391 (19.5)	52 (31.0)	
3	310 (14.3)	281 (14.0)	29 (17.3)	
4	216 (9.9)	204 (10.2)	12 (7.1)	
5	165 (7.6)	155 (7.7)	10 (6.0)	
6	395 (18.2)	368 (18.3)	27 (16.1)	
7	233 (10.7)	216 (10.8)	17 (10.1)	
8	46 (2.1)	43 (2.1)	3 (1.8)	

Numbers are no. (%) unless otherwise noted.

CABG: coronary artery bypass grafting; COPD: chronic obstructive pulmonary disease; eGFR: estimated glomerular filtration rate; ICD: implantable cardioverter defibrillator; LVEF: left ventricular ejection fraction; PCI: percutaneous coronary intervention; Q: quartile; SD: standard deviation.

### Sensitivity and subgroup analysis

Sensitivity analysis was performed using multivariable Cox regression for all outcomes. We also analysed patients with prosthetic and native valve endocarditis separately, as well as trends over time, comparing surgeries performed 1997–2005, 2006–2014, and 2015–2022. Furthermore, we conducted a sensitivity analysis excluding patients who died within 90 days of surgery, acting as a generous proxy for excluding patients with severely invasive disease who commonly die early after surgery, and finally, an analysis excluding concomitant valve surgery.

## RESULTS

### Baseline characteristics

Among 2175 patients who underwent aortic valve replacement for IE during the study period, 168 (8%) received a first-time permanent pacemaker or ICD within 30 days of surgery and 2007 (92%) did not. The mean age of the total study cohort was 59 years; 397 (18%) were female. Emergent surgery (within 24 hours from the decision to operate) was performed in 14% of patients, 27% had prior heart failure, and 7% had had a previous endocarditis episode. Most pacemakers (80%) were implanted with transvenous right heart chamber lead placement. Before weighting, patients in the pacemaker group were older (mean age 64 vs 59 years), more frequently had prosthetic valve endocarditis, and more often had left ventricular ejection fraction below 30% than patients in the no pacemaker group. Rates of multiple valve surgery and root replacement were also higher in the pacemaker group, but fewer patients in the pacemaker group had emergent surgery. Baseline characteristics according to pacemaker implantation status are shown in Table 1. Supplementary Material, Fig. S1, shows the absolute mean differences before and after weighting; the balance was achieved for all baseline characteristics. Patient enrolment according to calendar year is shown in Supplementary Material, Fig. S2, and the number of days until pacemaker implantation is shown in Supplementary Material, Fig. S3.

### Survival

**Figure 1:**
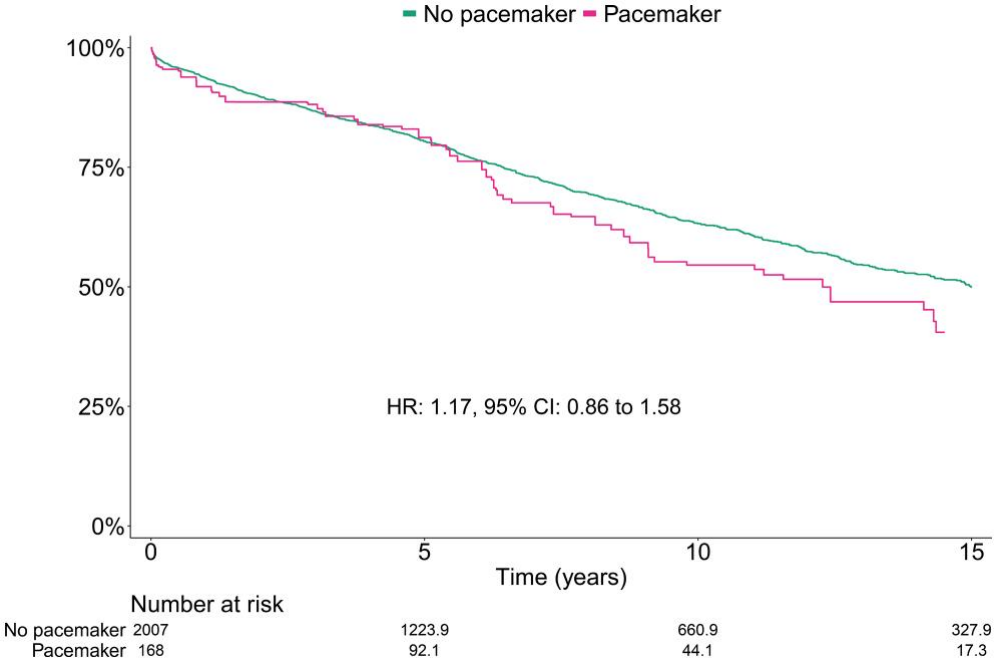
Kaplan–Meier estimated survival after aortic valve surgery for infective endocarditis, weighted population. CI: confidence interval; HR: hazard ratio.

During a median (95% CI) follow-up time of 7.8 (5.6–10.9) years in the pacemaker group (maximum 24.7 years) and 10.6 (9.6–11.2) years in the no pacemaker group (maximum 26.1 years), 65 (39%) and 757 (38%) patients died in the pacemaker and no pacemaker group, respectively. After weighting, the cumulative incidence of all-cause mortality in the pacemaker vs no pacemaker group at 1, 5, 10 and 15 years was 8%, 19%, 45% and 60% vs 6%, 20%, 37% and 50%, respectively. There was no statistically significant association between pacemaker implantation and death during follow-up (HR 1.17; 95% CI 0.86–1.58; *P* = 0.70). The survival curve for patients in the pacemaker vs no pacemaker group after weighting is presented in Fig. 1. Cumulative incidence rates of all-cause mortality after weighting are presented in Table 2.

**Table 2:** Cumulative incidence of all-cause mortality and, accounting for the competing risk of death, cumulative incidences of heart failure hospitalization and reinfection (% (95% CI))

	1 year	5 years	10 years	15 years
All-cause mortality				
No pacemaker	6.3 (5.2–7.4)	19.6 (17.7–21.4)	36.8 (34.2–39.2)	50.2 (47.1–53.0)
Pacemaker	8.2 (3.3–12.8)	18.8 (11.4–25.6)	45.4 (33.5–55.2)	59.5 (44.0–70.7)
Heart failure hospitalization				
No pacemaker	1.9 (1.5–2.5)	6.8 (5.8–7.9)	11.5 (10.1–13.1)	15.8 (14.0–17.7)
Pacemaker	2.7 (1.7–4.5)	9.4 (6.0–14.4)	15.4 (9.9–23.0)	20.6 (13.4–30.2)
Reinfection				
No pacemaker	4.2 (3.5–5.1)	11.0 (9.7–12.5)	15.3 (13.7–17.0)	18.2 (16.3–20.2)
Pacemaker	3.6 (2.1–6.0)	9.2 (5.5–14.9)	12.6 (7.6–20.1)	14.7 (8.9–23.3)

All results are after weighting. A description of the weighting procedure can be found in the statistical methods section.

CI = confidence interval.

### Heart failure

**Figure 2:**
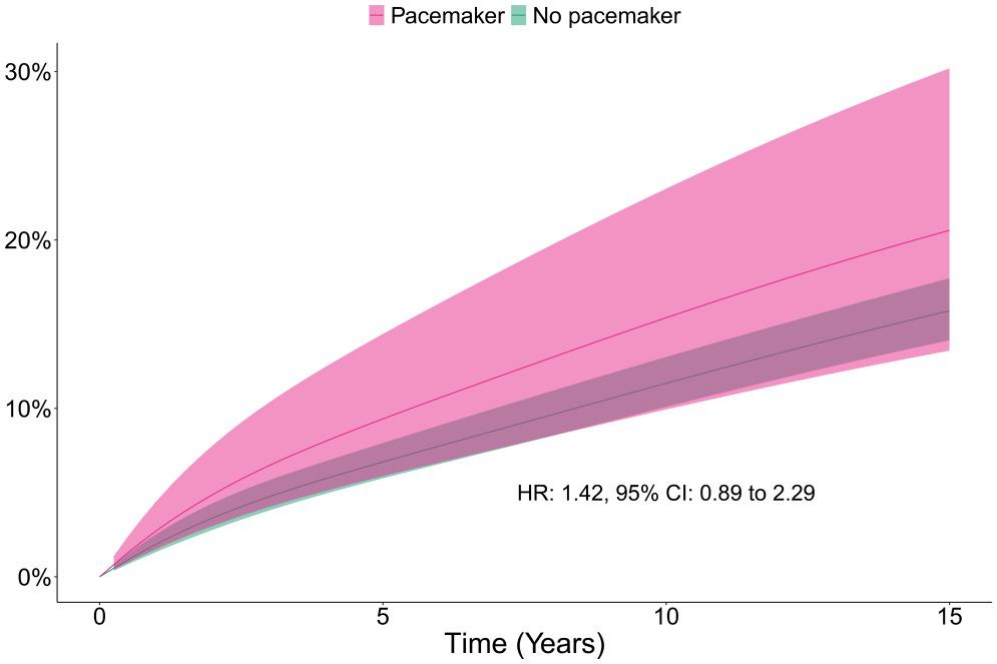
Cumulative incidence of heart failure hospitalization after aortic valve surgery for infective endocarditis, weighted population. CI: confidence interval; HR: hazard ratio.

During follow-up, 23 (14%) patients in the pacemaker group and 244 (12%) patients in the no pacemaker group were hospitalized for heart failure. After weighting, the cumulative incidence of heart failure hospitalization in the pacemaker versus no pacemaker group at 1, 5, 10 and 15 years was 3%, 9%, 15% and 21% vs 2%, 7%, 12% and 16%, respectively. There was no statistically significant association between pacemaker implantation and heart failure hospitalisation during follow-up (HR 1.42, 95% CI 0.88–2.29; *P* = 0.86). The cumulative incidences of heart failure are presented in Fig. 2. The cumulative incidence rates of heart failure after weighting are presented in Table 2.

### Reinfection

**Figure 3:**
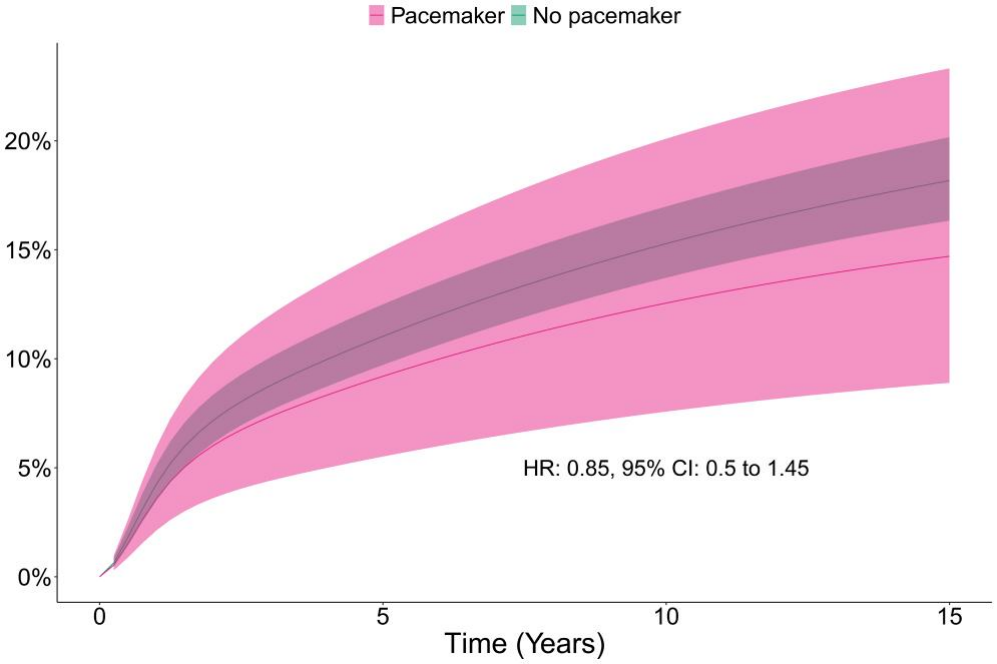
Cumulative incidence of reinfection after aortic valve surgery for infective endocarditis, weighted population. CI: confidence interval; HR: hazard ratio.

During follow-up, 16 (10%) patients in the pacemaker group and 294 (15%) patients in the no pacemaker group had reinfection. After weighting, the cumulative incidence of reinfection in the pacemaker vs no pacemaker group at 1, 5, 10 and 15 years was 4%, 9%, 13% and 15% vs 4%, 11%, 15% and 18%, respectively. There was no statistically significant association between pacemaker implantation and reinfection during follow-up (HR 0.85; 95% CI 0.50–1.45; *P* = 0.15). The cumulative incidences of reinfection are presented in Fig. 3. The cumulative incidence rates after weighting are presented in Table 2.

### Sensitivity and subgroup analyses

Multivariable Cox regression analysis yielded similar results to those of our primary analyses for all our outcomes. The trends over time analysis (Supplementary Material, Table S4) indicated no difference between the time periods. The remaining sensitivity analyses detailed above also yielded similar results to those of our primary analyses.

### Missing data

Data were missing for the following variables: left ventricular ejection fraction (14%), estimated glomerular filtration rate (7%) and body mass index (14%). Follow-up data were complete for all outcomes.

## DISCUSSION

In this large, nationwide, population-based study of surgically treated patients with aortic valve endocarditis, the rate of permanent pacemaker implantation was twice as high as seen after surgical aortic valve replacement for non-IE indications at the same institutions [9]. As expected, the patients who received pacemakers more often had root replacements and more had concomitant surgeries, implying a more invasive IE disease at the outset. After balancing for these baseline differences, pacemaker implantation was not associated with higher rates of mortality, heart failure, or reinfection.

There have been few studies of surgically treated IE patients, and, to our knowledge, none have focused on clinical outcomes after pacemaker implantation. The impact of postoperative permanent pacemaker placement after surgical aortic valve replacement in non-IE patients was investigated by Glaser *et al.* [9], who studied a similarly recruited Swedish registry population of 24 938 patients undergoing primary aortic valve replacement for non-IE indications in Sweden 1997–2018; a new permanent pacemaker was implanted postoperatively in 3.4% of the patients. Pacemaker implantation was associated with an increased all-cause mortality rate (HR 1.14; 95% CI 1.01–1.29; *P* = 0.03) and an increased heart failure rate (HR 1.58; 95% CI 1.31–1.89; *P* < 0.001), but not with an increased rate of prosthetic valve endocarditis. In our study, which examined surgery for an IE-indication only, we found no statistically significant increase in the risk of death or heart failure among patients who received a postoperative pacemaker (mortality: HR 1.17, 95% CI 0.86–1.58; heart failure: HR 1.42, 95% CI 0.89–2.29). It remains possible that we would have detected a significant difference in outcomes had we had a larger study population and more events. Therefore, our results must be interpreted with caution. At the same time, our results indicate that pacemaker-requiring conduction disturbances in IE patients may be less influential on long-term mortality and morbidity than other factors, such as the severity of the disease and baseline comorbidities.

Using a cohort of IE patients, Pericart *et al.* investigated long-term outcomes in patients with atrioventricular block, a common indication for permanent pacemaker in IE patients. Pericart *et al.* performed a single-institution, observational study, investigating long-term outcomes in 616 patients with IE between 1990 and 2012 (47% had surgery) [27]. The rate of atrioventricular block after surgical treatment was 8% (compared to 3% in the non-surgically managed patients) and atrioventricular block was associated with an increased rate of death during follow-up (HR 1.80; 95% CI 1.02–3.16; *P* = 0.04). Pericart *et al.* included IE of any valve, and the mean follow-up time of the study was shorter than in our study (4.8 vs 8.0 years). While surgery was associated with a lower mortality rate, the authors did not present the impact of atrioventricular block on mortality rates for the surgical subpopulation [27]. We present a larger study, with a more focused study population and longer follow-up, adding novel information on the long-term prognosis specifically on surgically treated patients with aortic valve endocarditis.

The average rate of pacemaker implantation in our study was lower than in previous studies on IE [6–8, 28]. Possible reasons for this finding include differing rates of prosthetic valve IE between studies and the fact that our study primarily analysed aortic valve disease, excluding cases of only tricuspid and mitral valve disease. Nonetheless, the rate of pacemaker implantation in our study was still double that of the study by Glaser *et al.* [9], which contained non-IE patients operated in the same institutions, during approximately the same period, which supports that patients with IE still face an added risk of permanent pacemaker implantation.

Pacemaker implantation is associated with several complications, both in the short and long terms. Pacemaker-induced cardiomyopathy, a reduction in left ventricular function in the setting of right ventricular pacing, may lead to inefficient contractile function, elevated filling pressures and maladaptive cardiac remodelling [29, 30]. Furthermore, pacemaker implantation has been associated with other lead-related complications, such as pocket infection, lead issues, and tricuspid valve issues [31]. Interestingly, prior research found that only 40–45% of patients after AVR (for all indications) were pacing-dependent a few years after surgery [32, 33], raising questions if some permanent pacemaker implantations after cardiac surgery could be avoided.

This study increases the understanding of what receiving a permanent pacemaker after surgery for IE implies. Although a new heart block suggests a more advanced disease, our results show that lifesaving surgery is not importantly jeopardised by the need for a pacemaker. Our study also has clinical significance in a broader, epidemiological perspective, and provides a basis that can serve as reference material for future studies investigating pacemaker-related differences following aortic valve replacement and in the context of IE.

### Strengths and limitations

The size of our study population, in the context of cohorts of IE patients, as well as the long and complete follow-up for all outcomes, constitute important strengths of this study. Endocarditis is a rare disease, and it is challenging to recruit patients prospectively for randomized controlled trials. Therefore, well-designed observational studies are important. The Swedish health registries, which are well-validated and contain extensive information allowed us to adjust for over 30 important baseline factors. Still, our study has several limitations. As discussed above, and despite the relatively large size of our cohort, it remains possible that the power in our study was too low to detect a significant difference between the groups. Additionally, while we had some data on pacemaker type, we did not have specific information on leadless pacemakers, but we know they were fewer than 8. We were also missing data on pre-existing conduction system disease, such as bundle branch block and atrioventricular block, infecting microorganisms, results of electro- and echocardiography, pacemaker implantation indication, pacing burden, and pacemaker dependency after surgery. Where possible, we used other data that we deemed could serve as a proxy for the missing information in the weighting model; for example, root replacement served as a proxy for a more complex infection.

We adjusted for the year of surgery to counter limitations due to the long enrolment period, such as possible unmeasured variation in the patient population and developments in treatment guidelines. Finally, the inherent limitations that come with an observational study including residual confounding due to unknown and unmeasured confounding are inevitable. Nonetheless, the fact that we achieved balanced weighting between the groups strengthens the robustness of the results.

## CONCLUSION

We did not find that receiving a pacemaker after surgery for aortic valve endocarditis was associated with increased rates of all-cause mortality, heart failure, or reinfection. Pacemaker implantation is common in aortic valve IE patients and, although pacemaker need suggests more advanced disease, these results show that it does not importantly jeopardise lifesaving surgery for IE.

## Supplementary Material

ezaf125_Supplementary_Data

## Data Availability

The data underlying this article cannot be shared publicly due to data protection regulations; requests should be made to the corresponding author.

## ABBREVIATIONS

HR: Hazard ratio
ICD: Implantable cardioverter defibrillator
IE: Infective endocarditis
