# Supplementary material for: Implications of pacemaker implantation after aortic valve surgery for endocarditis: a nationwide study
Source: Eur J Cardiothorac Surg. 2025 Apr 8;67(4):ezaf125. doi: 10.1093/ejcts/ezaf125 (PMC12033029; doi:10.1093/ejcts/ezaf125)
Supplement: ezaf125_Supplementary_Data [file ezaf125_supplementary_data.docx]

**Supplemental Material**

Implications of Pacemaker Implantation After Aortic Valve Surgery for Endocarditis: a SWEDEHEART Study

Lisa O.F. Bearpark, MD^1^, Michael Dismorr, MD, PhD^1,2^, Anders Franco-Cereceda, MD, PhD^1,2^, Ulrik Sartipy, MD, PhD^1,2^, Natalie Glaser, MD, PhD^1,3^

^1^Department of Molecular Medicine and Surgery, Karolinska Institutet, Stockholm, Sweden

^2^Department of Cardiothoracic Surgery, Karolinska University Hospital, Stockholm, Sweden

^3^Department of Cardiology, Stockholm South General Hospital, Stockholm, Sweden

|  |  |  |
| --- | --- | --- |
| **sTable 1.** **Definition of outcomes.** Primary diagnosis from the National Patient Register. | | |
|  | ICD-9 codes | ICD-10 codes |
| Heart failure | 428 | I50 to I50.9 |
| Endocarditis | 421 | I33.0, I33.9, I38.9, I39.8, b376, T826, T827 |

| **sTable 2**. **Definition of diagnoses/comorbid conditions.** All diagnoses from the National Patient Register from 1987 and onward. | | |
| --- | --- | --- |
|  | ICD-9 codes | ICD-10 codes |
| Myocardial infarction | 410 | I21 to I21.9 |
| Stroke | 430 to 438 | I60 to I69.9 |
| Heart failure | 425, 428 | I11.0, I13.0, I13.2, I25.5, I42 to 142.9, I43 to I43.9, I50 to I50.9, K761 |
| Atrial fibrillation | 427D | I48 to I48.9 |
| Chronic obstructive pulmonary disease | 490 to 496 | J44 to J44.9 |
| Hypertension | 401 to 405 | I10 to I15.9 |
| Hyperlipidemia | 272 | E78 to E78.9 |
| Peripheral vascular disease | 440 to 446 | I65 to I65.9, I71 to I71.9, I73.8, I73.9 |
| Alcohol dependence | 291, 303, 571 | F10 to F10.9, K70 to K70.9 |
| Liver disease | 570 to 573 | K70 to K77.9 |
| Cancer | 140 to 208 | C00 to C97.9 |
| Endocarditis | 421 | I33.0, I33.9, I38.9, I39.8 |
| Diabetes mellitus  Freestyle or homograft prosthesis | 250 | E10 to E14.9  FMD30, FMD33 |
| Prior bleeding event | 285B, 430, 431, 432, 456A, 530H, 531A, 531C, 531E, 531G, 532A, 532C, 532E, 532G, 533A, 533C, 533E, 533G, 534A, 534C, 534E, 534G, 569D, 578. | D629, I60, I61, I62, I850, K226, K250, K252, K254, K256, K260, K262, K264, K266, K270, K272, K274, K276, K280, K282, K284, K286, K290, K625, K920, K921, K922 |

| **sTable 3**. **Definition of pacemaker diagnosis and patient distribution among 118 patients who received a new pacemaker, according to NOMESCO^1^ Classification of Surgical Procedures (NCSP)^2^.** | | |
| --- | --- | --- |
|  | NCSP codes | Number  of patients |
| Transvenous cardiac pacemaker with ventricular electrode | FPE00 | 22 |
| Transvenous cardiac pacemaker with atrial electrode | FPE10 | 3 |
| Transvenous cardiac pacemaker with atrial and ventricular electrode | FPE20 | 135 |
| Transvenous cardiac pacemaker with biventricular electrodes | FPE26 | 8 |
| Transvenous cardioverter-defibrillator with generator and ventricular electrodes | FPG30 | 2 |
| Transvenous cardioverter-defibrillator with generator and atrial and ventricular electrodes | FPG33 | 1 |
| Transvenous cardioverter-defibrillator with generator and biventricular electrodes | FPG36 | 2 |
| Unknown |  | 9 |
| \| ^1^NOMESCO = Nordic Medico-Statistical Committee. ^2^Codes FPF00, FPF10, FPF20, FPG10, FPG20 are not listed because they either did not have any patient tied to them or the patient had several codes attached and therefore was put in the “unknown” category. \| \| --- \| | | |

**sFigure 1. Covariate balance between groups before and after inverse probability of treatment weighting.**

**sFigure 2. Patient enrollment according to calendar year**

**sFigure 3. Days until pacemaker implantation**

| **sTable 4.** **Relative hazards for all-cause mortality, heart failure, and prosthetic valve endocarditis, using multivariable Cox regression, for patients depending on the surgery period**. | | | | |
| --- | --- | --- | --- | --- |
|  | **Period of surgery**  **1997-2005** | **Period of surgery**  **2006-2014** | **Period of surgery**  **2015-2022** |  |
|  | HR (95% CI) | HR (95% CI; P-value) | HR (95% CI; P-value) |  |
| All-cause mortality^a^ | Reference | 0.95  (0.74-1.22; 0.70) | 0.88  (0.64-1.21; 0.44) |  |
| Heart failure hospitalization^a^ | Reference | 0.73  (0.48-1.13; 0.15) | 0.81  (0.48-1.37; 0.43) |  |
| Prosthetic valve endocarditis^a^ | Reference | 0.80  (0.53-1.22; 0.31) | 0.90  (0.56-1.44; 0.66) |  |
| CI = confidence interval; HR = hazard ratio. ^a^Multivariable adjustment was made for all the factors included in sFigure 1. | | | |  |
